# Case report: A case of ocular infection caused by *Corynespora cassiicola*

**DOI:** 10.3389/fcimb.2023.1160831

**Published:** 2023-06-28

**Authors:** Qin Wang, Lu Wang, Lisha Lian, Xiaofeng Pu, Lu Tang, Yanmei Li, Yuan Liu

**Affiliations:** Clinical Laboratory, The General Hospital of Western Theater Command, Chengdu, China

**Keywords:** ocular infection, *Corynespora cassiicola*, morphology, pseudospores, drug sensitivities

## Abstract

**Objective:**

The aim of this study is to identify the pathogen causing ocular infection in a Chinese patient and to describe its morphological characteristics.

**Methods:**

Samples from the patient’s intraoperative pus were collected for microscopic examination and culture. Morphology and drug sensitivities of the isolated fungus were analyzed. Ribosomal DNA (rDNA) sequencing was performed and blasted in GenBank.

**Results:**

A strain of fungi was repeatedly isolated from pus samples in different types of medium. No conidia were shown when the isolate cultured on normal PDA medium, whereas pseudoseptate thick-walled conidia were shown when cultured on medium containing leaf leachate. The results of BLAST and phylogenetic trees based on internal transcribed spacer, beta-tubulin, translation elongation factor 1-alpha, and RNA polymerase II gene demonstrated that the isolated fungus was *Corynespora cassiicola*. Minimum inhibitory concentration results of this organism were as follows: anidulafungin, 0.06 μg/ml; amphotericin B, 0.12 μg/ml; micafungin, 0.06 μg/ml; caspofungin, 0.5 μg/ml; 5-fluorocytosine, >64 μg/ml; posaconazole, 2 μg/ml; voriconazole, 0.25 μg/ml; itraconazole, 0.5 μg/ml; fluconazole, 64 μg/ml.

**Conclusion:**

The case was infected with *Corynespora cassiicola* and led to eye suppurative endophthalmitis and blindness. Combined applications of morphological and molecular biology techniques facilitate accurate diagnosis of fungal infections.

## Introduction

*Corynespora* is widely distributed in nature, and most of them are parasitic fungi, which exist mostly on the stem, branches, leaves, or topsoil of plants in tropical and subtropical regions. *Corynespora* mainly causes leaf spot disease in cucumber, cowpea, banana, and other plants (Zhang, 2018), whereas infection in humans is rare (Lopez et al., 2018). In this paper, we report a case of severe infection caused by *Corynespora cassiicola* (*C. cassiicola*) that resulted in evisceration of the eye contents.

## Case presentation

This patient was a 66-year-old male farmer, who suffered from left eye swelling and pain. Six months ago, the patient developed mild blurred vision with a small amount of eye discharge. Twenty days before presenting to the hospital, the patient developed the sudden onset of swelling, pain, and blurred vision in the left eye. He was treated with intravenous amoxicillin/clavulanic acide at a local hospital but had progression of the blurred vision and distension. The patient had a history of hypertension for four years and was taking amlodipine benzoate.

**Figure 1:**
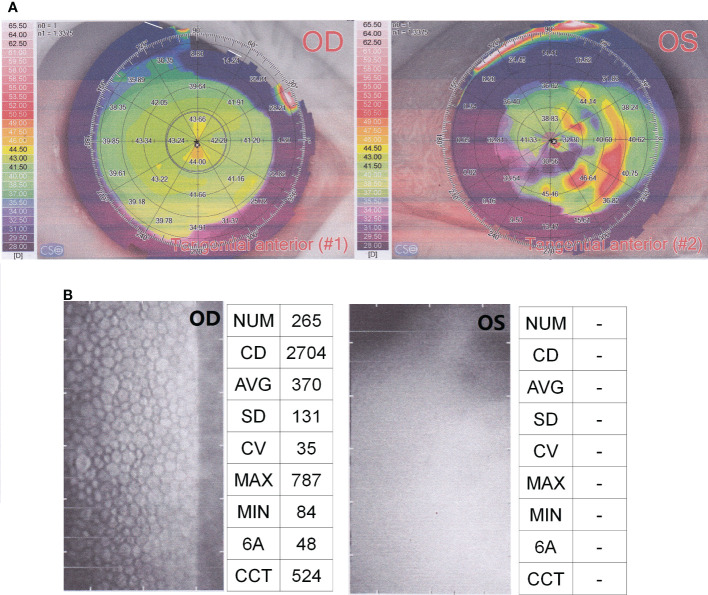
Results of corneal topography **(A)** and corneal endothelial cell quality detection **(B)**. OD, OculusDexter, representing the right eye; OS, OculusSinister, representing the left eye.

Physical examination: There was no light perception in the left eye. Conjunctival hyperemia and corneal edema were evident. A large amount of flocculent milky white pus was seen in the anterior chamber. The texture of the iris could not been clearly seen via slit lamp examination. The examinations of corneal topography showed an abnormal corneal curvature of left eye (**Figure 1A**). The corneal endothelial cell quality detection showed that almost no intact corneal cells could be seen in the left eye (**Figure 1B**). These results meant that the cornea and eyeball of left eye were almost completely destroyed (**Figure 1**).

The diagnosis was left eye endophthalmitis and left eye blindness, accompanied with progressive worsening of symptoms and lack of ocular preservation value. Left eye evisceration was performed. A large amount of milky white purulent flocs was seen during the operation, and the pus in the anterior chamber was taken for microbial smear and culture. Clear mycelium with septa and branches were observed in smear test, and grayish-white fluffy colonies were cultured in different types of medium. After surgery, erythromycin eye ointment and levofloxacin eye drops were given to control infection. The patient recovered well after surgery and was discharged from hospital on 10 January 2022. No antifungal drugs were given. No abnormalities were found during follow-up.

## Laboratory investigations

### Samples source

Pus samples were taken from intraoperative milky white purulent flocculent.

### Natural substrate medium

Dry leaves were cut into pieces and soaked in distilled water for 6 h and then boiled and centrifuged at 2,500 rpm for 10 min. The supernatant was filtered through a 0.22-μm sterilization filter. Natural substrate potato dextrose agar (PDA) agar medium was prepared using 20 g of dry powder of PDA medium (Beijing Tiantan Bio) dissolved in 1,000 ml of supernatant of distilled water soaked in dry leaves (Zhang, 2018). Meanwhile, ordinary PDA agar medium was prepared using distilled water.

### Molecular biology identification

Fungal DNA extraction kit (EE101, TransGen Biotech, Beijing, China) was used to extract DNA of the isolates. Internal transcribed spacer (ITS), beta-tubulin (BT2), translation elongation factor 1-alpha (tef-1), and RNA polymerase II (RPB2) gene were amplified using PCR as follows. PCR reaction system: Taq of 0.5 μl, 2× Taq Master Mix of 25 μl, deoxynucleotide triphosphates (dNTPs) of 5 μl, upstream and downstream primers of 0.5 μl each, template DNA of 2 μl, and ddH_2_O of up to 50 μl. Reaction conditions: pre-denaturation at 94°C for 2 min; denaturation at 94°C for 30 s, annealing at 58°C for 30 s, extension at 72°C for 90s, cycle number 35; extension at 72°C for 7 min. The primer sequences (5′-3′) were ITS1: TCCGTAGGTGAACCTGCGG and ITS4: TCCTCCGCTTATTGATATGC for ITS (White et al., 1990); T1: AACATGCGTGAGATTGTAAGT and Bt2b: ACCCTCA-GTGTAGTGACCCTTGGC for BT2 (Rezaei-Matehkolaei et al., 2014); EF1-1018F: GAYTTCATCAAGAACATGAT and EF1-1620R: GACGTTGAADCCRACRTTGTC for tef-1 (Raja et al., 2017); DRPB2-5F: GAYACNGAYGAYCGWGAYCAYTTYGG and DRPB2-7R: AANCCCATDGCYTGYTTDCCCAT for RPB2 (Voglmayr et al., 2020). The PCR products were identified by 1% agarose gel electrophoresis and sequenced by Tsingke Biotechnology Co (Chengdu, China).

The sequences were submitted to GenBank, and accession numbers were obtained (OQ658167 for ITS, OQ700917 for BT2, OQ700916 for tef-1, and OQ700915 for RPB2). Phylogenetic trees of ITS, tef-1, and RPB2 were constructed by neighbor-joining and maximum likelihood methods with 1,000 bootstrap replicates using MEGA 6.06 software.

### Antifungal susceptibility test

Antifungal susceptibility tests were performed by using the Sensititre YeastOne™ YO10 (CN15009V2, Thermo Scientific, Cleveland, OH, USA) according to the manufacturer’s instructions. Briefly, the conidia were collected with a cotton swab, suspended in a sterile saline solution containing Tween-20, and inoculated on the drug-sensitive plate. The broth microdilution panel was incubated in a non-CO_2_ incubator at 35°C for 72 h. *Candida albicans* ATCC14053 was used as the quality control strain in this test. The minimum inhibitory concentration (MIC) values were read according to the MIC interpretation standard of *Aspergillus* following the kit instructions (recommended by CLSI M38 and EUCAST).

## Pathogenic results

### KOH fungal smear test

Intraoperative pus samples were examined by microscopy with 10% KOH. Clear mycelium with septa and branches were observed (**Supplementary Figure 1**).

### Fungal culture

**Figure 2:**
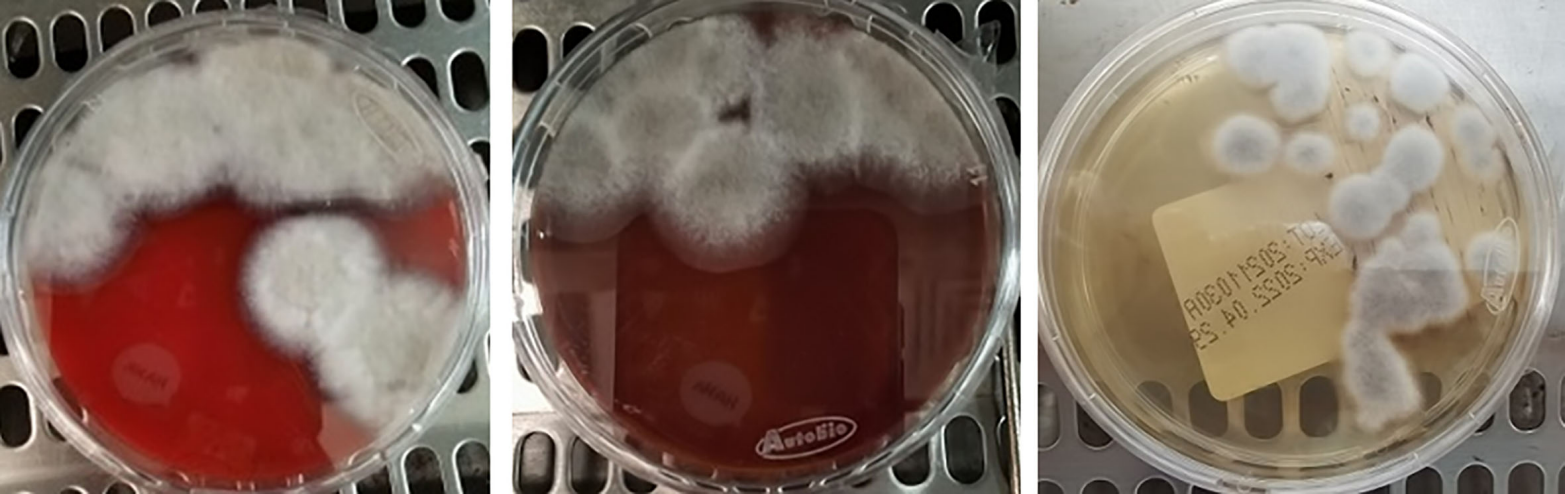
Colony morphology in blood medium (left), chocolate medium (middle), and Sabouraud’s medium (right) at 28°C for 3 days.

The pus specimens were inoculated aseptically with blood medium, chocolate medium, and Sabouraud’s medium. After 3 days of incubation, grayish-white fluffy colonies were observed in all three types of medium (**Figure 2**). By lactic acid phenol cotton blue staining, a large number of mycelium were observed, and no conidia were seen.

The mycelium was transferred to Sabouraud’s medium and PDA medium, and incubated at 28° and 36°C. This strain grew better at 28°C than at 36°C, and a large number of racket-like mycelium could be seen in lactophenol cotton blue staining, as shown in **Supplementary Figure 2**. However, no conidia were found in the culture up to 40 days. It has been reported that some of the *Corynespora* is not easy to sporulation *in vitro* culture, and the medium composition should be as close to the natural substrate as possible, such as adding wood chips or crushed sticks (Zhang, 2018).

**Figure 3:**
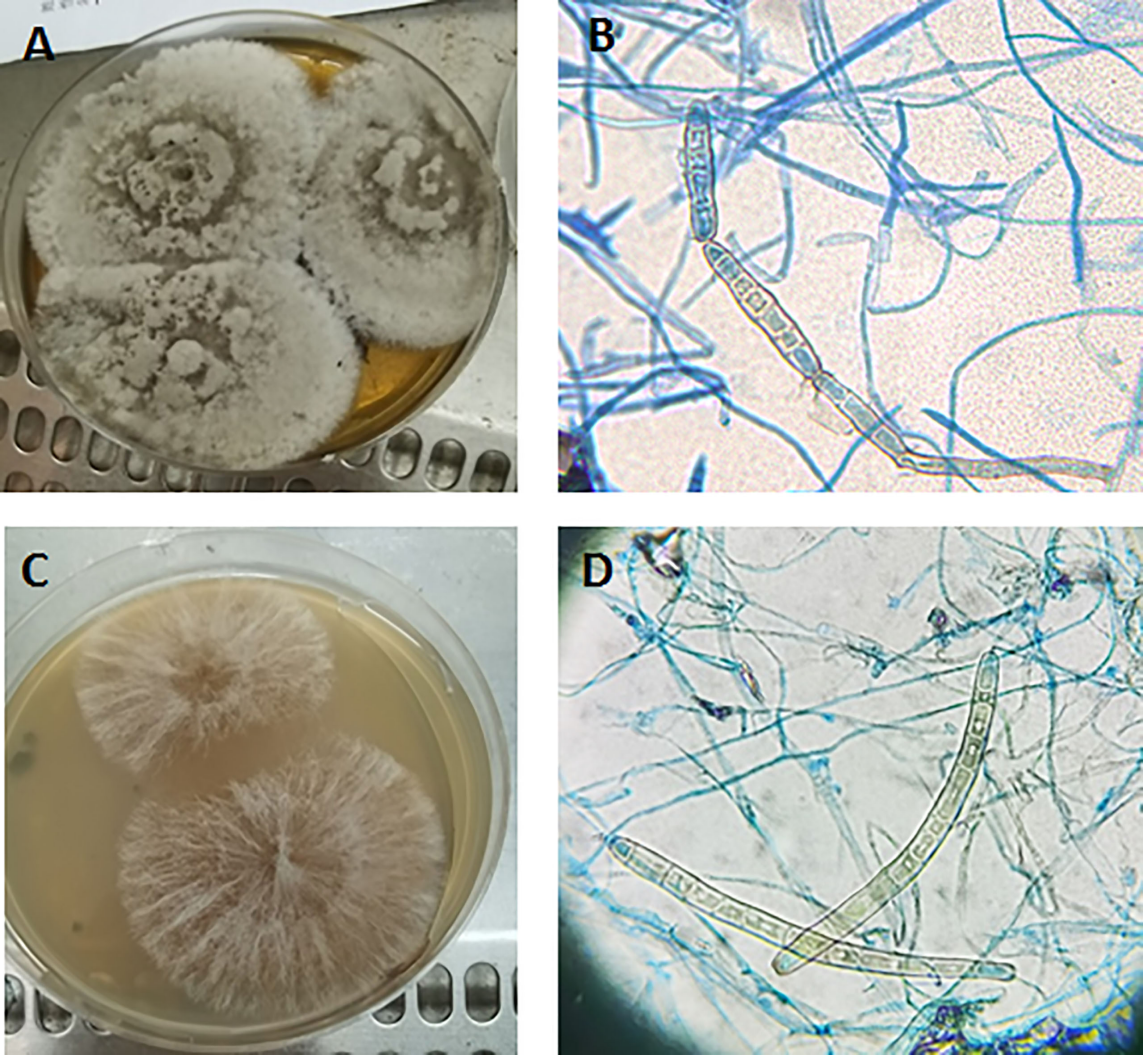
Colony morphology **(A)** and conidia **(B)** on PDA medium containing leaf leachate incubated at 28°C for 5 days. Colony morphology **(C)** and conidia **(D)** on water plate containing leaf leachate incubated at 28°C for 7 days **(B, D)** 40×.

Natural substrate medium was prepared as described above. The strain was transferred to four types of plates: normal PDA medium, PDA medium with leaf leachate, normal water plate, and water plate with leaf leachate. The colonies on PDA medium containing leaf leachate grew more rapidly than those of normal PDA medium, and pseudospores were seen in chains or scattered at 5 days of incubation (**Figures 3A, B**). The colonies on water plate containing leaf leachate were sparsely downy at 7 days of incubation, pseudospores with thick-walled conidia appeared, and most of the conidia were scattered individually (**Figures 3C, D**). Colonies on normal PDA medium and water plate grew slowly, and no conidia were seen.

### Molecular biological identification

**Figure 4:**
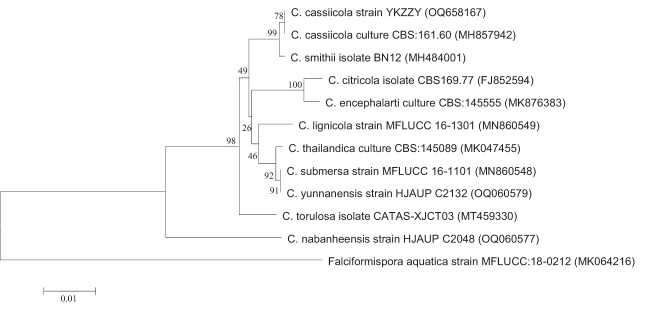
Phylogenetic analysis of ITS. Phylogenetic trees were constructed by neighbor-joining method with 1,000 bootstrap replicates. The tree is rooted with *Falciformispora aquatica* strain MFLUCC:18-0212 as outgroup.

The ITS, BT2, tef-1, and RPB2 sequences of this strain were obtained and compared using the on line BLAST program (NCBI Blastn). All the sequencing data showed a high identity to that of *C. cassiicola* (**Supplementary Table 1**). Phylogenetic trees based on ITS, tef-1, and RPB2 also indicated that the isolated strain of this case belonged to the species *C. cassiicola* (**Figure 4**; **Supplementary Figure 3**). Phylogenetic trees constructed by maximum likelihood method displayed a consistent result (data not shown).

### *In vitro* antifungal susceptibility testing

Sensititre YeastOne™ YO10 was used to detect the MIC of the fungus. Susceptibility results are as follows: anidulafungin, 0.06 μg/ml; amphotericin B, 0.12 μg/ml; micafungin, 0.06 μg/ml; caspofungin, 0.5 μg/ml; 5-fluorocytosine, >64 μg/ml; posaconazole, 2 μg/ml; voriconazole, 0.25 μg/ml; itraconazole, 0.5 μg/ml; fluconazole, 64 μg/ml.

## Discussion

*C. cassiicola* belongs to the order Pleosporales and genus *Corynespora*, which causes target leaf spot disease worldwide (Crous et al., 2020). *C. cassiicola* and *C. smithi* form a specific family within Pleosporales (Schoch et al., 2009). Most species of the *Corynespora* genus are anamorphic and *C. Cassiicola* is highly genetically diverse, whereas the relationship between phylogenetic lineages and pathogenicity is not clear (Lopez et al., 2018).

*C. cassiicola* infections are rare in humans. A total of 14 cases have been reported from 2010 to present (**Supplementary Table 2**). These cases were mostly skin soft tissue infections. Four cases were ocular infections reported in India, Korea, Malaysia, and Japan (Yamada et al., 2013; Looi et al., 2017; Chung et al., 2018; Gupta et al., 2022). One case was intracerebral infection reported in China (Song et al., 2022). Several cases were associated with risk factors for fungal infections, such as malnutrition, long-term corticosteroid inhalation, and diabetes (Huang et al., 2010; Xie et al., 2018; Wang et al., 2019). Studies have shown that *C. cassiicola* has the potential to be vascular invasive and may lead to disseminated infections in immunodeficient hosts (Lv et al., 2011). Three cases of CARD9 deficiency causing infection with this fungus had been reported, and the treatment of antifungal infection was ineffective in two of them (Yan et al., 2016; Arango-Franco et al., 2018; Wang et al., 2022). Notably, most cases were from Asia. Several studies revealed that isolates from the different hosts were genetically distinct and host specialized (Dixon et al., 2009; Sumabat et al., 2018). In the future, more attention should be paid to the differences of genetic evolution between human and plant isolates as well as the genetic characteristics of human susceptible strains.

In this case, the patient had been farming cucumbers, tomatoes, radishes, and other crops for a long time. Farming may increase the risk of ocular exposure to *Corynespora* by rubbing eyes after contact with vegetation. The specimen obtained from intraoperative sampling revealed a large number of mycelium by direct microscopic examination. The same fungus grew in different types of medium after specimen inoculation, suggesting that the patient was infected by this *Corynespora*. Unfortunately, because of the patient’s delay in presentation and lack of antifungal therapy, the left eyeball was completely destroyed by the time of admission, with the serious consequence of ocular blindness.

The main morphological characteristics of *Corynespora* are conidia with solitary, paired chains, and false septa, and sporogenous cells are monoporous, synphytic, terminal, finite, or outspread from the top layer (Zhang, 2018). There was lack of conidiation when the isolate was cultured on PDA and normal water medium. Pseudoseptate thick-walled conidia were shown when cultured on medium containing leaf leachate. Because of the conidia of this train, it is probably more likely to be formed on the medium with dead wood degradation component when cultured *in vitro*, which provides a methodological reference for the culture of similar strains. Gene sequence analysis provides a powerful tool for fungal species identification, especially molds that fail to sporulate, which were impossible to speciate. Some clinical laboratories use DNA sequencing as part of routine protocols for fungal identification. We obtained the ITS, BT2, tef-1, and RPB2 sequences of this strain and constructed phylogenetic trees. All the sequences of this strain were submitted into GenBank and had a high similarity with that of *C. cassiicola*.

There are no breakpoints for this organism according to CLSI or EUCAST. MIC values to antifungal agents for this organism were reported in four publications (**Supplementary Table 3**). Because of the lack of standardized drug sensitivity test methods and breakpoints for *C. cassiicola*, the extent to which MIC values reflecting drug sensitivity still needs to be further clarified. There are no specific therapy recommendations for this organism. Voriconazole (VRC) and amphotericin B (AMB) were the most frequently used agents against this organism, which resulted in successful treatment outcomes in most cases (**Supplementary Table 2**). Terbinafine (TBF) was successfully used in two cases (nos. 6 and 13 in **Supplementary Table 2**).

However, two patients with CARD9 mutations (case nos. 9 and 11 in **Supplementary Table 2**) did not respond well (Yan et al., 2016; Arango-Franco et al., 2018). Three cases of eye infection (case nos. 2, 7, and 12 in **Supplementary Table 2**) used systemic and/or topical VRC. One (case no. 7 in **Supplementary Table 2**) additionally used amphotericin B injected into the anterior chamber (Chung et al., 2018), and one (case no. 12 in **Supplementary Table 2**) additionally combined application of micafungin and pimaricin ointment (Yamada et al., 2013). Clinical symptoms improved in all three cases. Notably, the penetration ability of antifungal drugs should be considered in case of eye infection, as echinocandins lack of penetration into vitreous.

## Data availability statement

The raw data supporting the conclusions of this article will be made available by the authors, without undue reservation.

## Ethics statement

The studies involving human participants were reviewed and approved by The ethics committee of General Hospital of Western Theater Command. Written informed consent for participation was not required for this study in accordance with the national legislation and the institutional requirements. Written informed consent was obtained from the participant/patient(s) for the publication of this case report.

## Author contributions

YLiu: Designed the study and wrote the paper. QW, LW, LL, XP, LT, and YLi: Contributed in experimental work. YLiu and QW: Analyzed the data. All authors contributed to the article and approved the submitted version.
