# Supplementary material for: Case report: A case of ocular infection caused by Corynespora cassiicola
Source: Front Cell Infect Microbiol. 2023 Jun 28;13:1160831. doi: 10.3389/fcimb.2023.1160831 (PMC10338080; doi:10.3389/fcimb.2023.1160831)
Supplement: Supplementary file 1 [file DataSheet_1.docx]

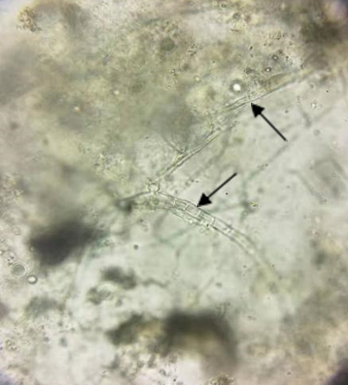


**Supplement figure 1** Mycelium detected by 10% KOH smear (40×)


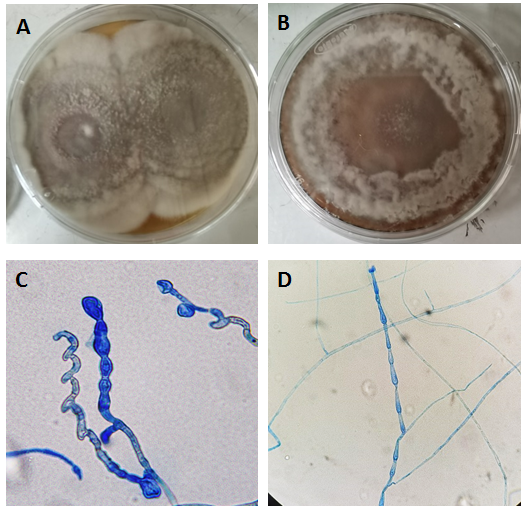


**Supplement figure 2** Colony morphology on PDA medium (A) and Sabouraud's medium (B) incubated at 28℃ for 6 days. Racquetted mycelium was observed after 6 days of incubation (C:PDA medium; D:Sabouraud's medium; 40×).


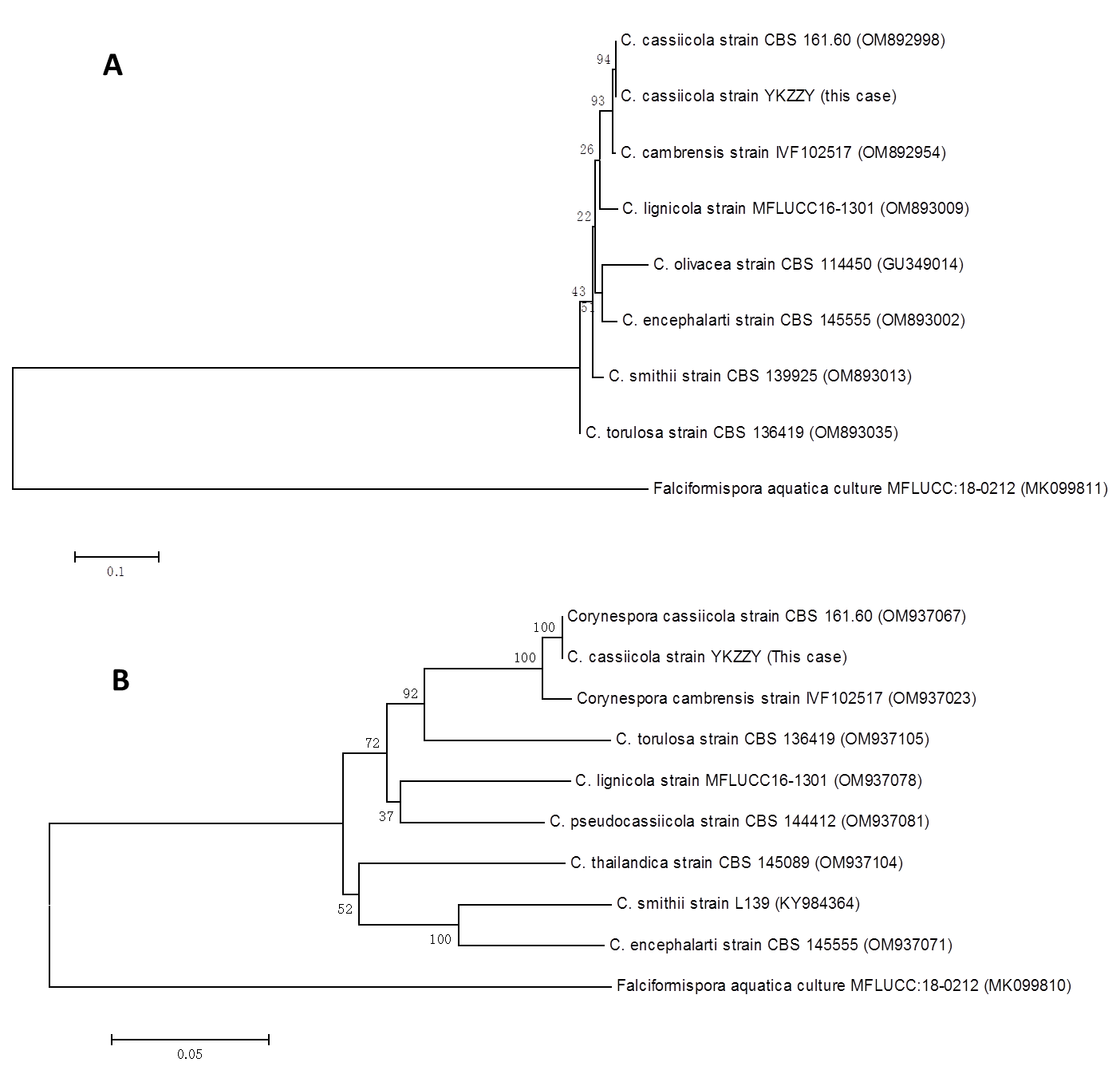


**Supplement figure 3** Phylogenetic analysis of tef-1 (A) and RPB2 (B). Phylogenetic trees were constructed by neighbor-joining method with 1000 bootstrap replicates. The tree is rooted with *Falciformispora aquatica* strain MFLUCC:18-0212 as outgroup.

**Supplement table 1**. Results of sequences blast in GenBank.

| Sequences of this case (accession No.s) | Sequences in GenBank (accession No.s) | Identity |
| --- | --- | --- |
| ITS (OQ658167) | *Corynespora cassiicola* strain GXCc6 internal transcribed spacer 1, partial sequence; 5.8S ribosomal RNA gene and internal transcribed spacer 2, complete sequence; and large subunit ribosomal RNA gene, partial sequence (MW300946) | 100% |
| BT2 (OQ700917) | *Corynespora cassiicola* isolate NC-1 beta-tubulin gene, partial cds (MZ337817) | 99.7% |
| tef-1 (OQ700916) | *Corynespora cassiicola* strain CBS 161.60 translation elongation factor 1-alpha (tef1) gene, partial cds (OM892998) | 100% |
| RPB2 (OQ700915) | *Corynespora cassiicola* isolate CD09101704 RNA polymerase II second largest subunit (RPB2) gene, partial cds (OL439938) | 100% |

**Supplement table 2**. Review of cases infected by C.cassiicola.

| **No.** | **Age** | **Sex** | **Location** | **Infection type** | **Area** | **Predisposing Cause** | **Basic illness** | **Treatment** | **Identify** | **References** |
| --- | --- | --- | --- | --- | --- | --- | --- | --- | --- | --- |
| 1 | 54 | M | right lower extremity | ulcerative lesion | China | skin trauma | immunosuppressive treatment for nephrotic syndrome | Aminolevulinic acid- photodynamic therapy | ITS | Feng et al. 2023 |
| 2 | 54 | M | right eye | endophthalmitis | India | Intravitreal triamcinolone acetonide injection | non-proliferative diabetic retinopathy and macular oedema | VRC | ITS | Gupta et al. 2022 |
| 3 | 76 | M | right leg | Subcutaneous phaeohyphomycosis | China | NA | none | FCA  VRC | ITS | Zou et al. 2022 |
| 4 | 68 | M | left upper extremity | deep dermatophytosis | China | NA | Pulmonary Cladosporium infection & *CARD9* mutation | VRC | morphological characteristics | Wang et al. 2022 |
| 5 | 22 | M | Cerebrospinal fluid | Infratentorial subdural effusion | China | NA | coma and progressive cerebellar herniation | AMB  VRC | NGS | Song et al. 2022 |
| 6 | 84 | M | right hand | subcutaneous infection | China | NA | COPD | ITC  VRC  TBF | ITS | Wang et al. 2019 |
| 7 | 74 | M | cornea | keratitis | Korea | sewage | NA | VRC  AMB | ITS | Chung et al. 2018 |
| 8 | 76 | M | right leg | Phaeohyphomycosis | China | NA | COPD & hypertension | VRC | ITS | Xie et al. 2018 |
| 9 | 8 | F | face | destructive mucocutaneous infection | Colombia | none | *CARD9* deficiency | AMB  VRC  POS  TBF | ITS | Arango-Franco et al. 2018 |
| 10 | NA | NA | contact lens | keratomycosis | Malaysia | NA | NA | NA | ITS & genome analysis | Looi et al. 2017 |
| 11 | 37 | F | face | subcutaneous phaeohyphomycosis | China | NA | *CARD9* mutation | AMB | ITS | Yan et al. 2016 |
| 12 | 76 | M | cornea | keratitis | Japan | plant | NA | VRC | ITS | Yamada et al. 2013 |
| 13 | 57 | F | right leg | subcutaneous phaeohyphomycosis | China | plant | NA | TBF | ITS | Lv et al. 2011 |
| 14 | 69 | F | upper limbs | subcutaneous infection | China | plant | diabetes mellitus & Iatrogenic Cushing syndrome | AMB | ITS | Huang et al. 2010 |

ITS: Internally Transcribed Spacer; ITC: itraconazole; POS: posaconazole; TBF: terbinafine; AMB: amphotericin B; VRC: voriconazole; FCA: Fluconazole; NGS: next-generation sequencing; CARD9: caspase-associated recruitment domain (CARD)-containing protein 9; COPD: Chronic obstructive pulmonary disease. NA: not applicable.

**Supplement table 3**. Minimum inhibitory concentrations (MICs) to antifungal drugs of *C.cassiicola*.

| References | polyene ntibiotics | | allylamine class | | azole derivatives | | | | | | | echinocandin class | | | other |
| --- | --- | --- | --- | --- | --- | --- | --- | --- | --- | --- | --- | --- | --- | --- | --- |
|  | AMB | NYT | TBF | BTF | VRC | ITC | POS | FCA | KCZ | ECZ | BFZ | MCF | ANF | CSF | 5-FC |
| Feng et al. 2023 | 0.25 | NA | <0.03 | NA | 0.25 | 1 | NA | NA | NA | NA | NA | 4 | NA | NA | NA |
| Arango-Franco et al. 2018 | 0.125 | NA | NA | NA | 0.75 | NA | 1 | NA | NA | NA | NA | NA | NA | NA | NA |
| Lv et al. 2011 | NA | 1 | 0.125 | 0.25 | 8 | NA | NA | 8 | 1 | 2 | 16 | NA | NA | NA | NA |
| Huang et al. 2010 | 0.5 | NA | NA | NA | 1 | NA | NA | NA | NA | NA | NA | NA | NA | NA | NA |
| This paper | 0.12 | NA | NA | NA | 0.5 | NA | 2 | 64 | NA | NA | NA | 0.06 | 0.06 | 0.5 | > 64 |

AMB: amphotericin B; NYT: nystatin; TBF: terbinafine; BTF:butenafine; VRC: voriconazole; ITC: itraconazole; POS: posaconazole; FCA: fluconazole; KCZ: ketoconazole; ECZ:econazole; BFZ: bifonazole; MCF: micafungin; ANF:anidulafungin; CSF:caspofungin; 5-FC:5-fluorocytosine. NA: not applicable.

**References**

Feng, Y., Zeng, Q., Qiu, Y., Li, D., and Shi, D. (2023). Successful application of photodynamic therapy for skin infection caused by corynespora cassiicola in an immunosuppressed patient and literature review. Photodiagnosis Photodyn. Ther. 41, 103279. doi: 10.1016/j.pdpdt.2023.103279

Zou, J. J., Li, J., Ma, S. S., Li, P. F., and Zhou, D. H. (2022). Subcutaneous phaeohyphomycosis caused by plant pathogenic corynespora cassiicola: a case report. Chin. J. Traumatol. 25 (6), 400–403. doi: 10.1016/j.cjtee.2022.09.002
